# The effect of an arm sling on energy consumption while walking in hemiplegic patients: a randomized comparison

**DOI:** 10.1177/0269215510381167

**Published:** 2011-01

**Authors:** Seung Hoon Han, Taikon Kim, Seong Ho Jang, Mi Jung Kim, Si-bog Park, Seoung Ic Yoon, Bong-Kun Choi, Michael Y Lee, Kyu Hoon Lee

**Affiliations:** Department of Rehabilitation Medicine, Hanyang University, Seoul; Department of Preventive Medicine, Kyunghee University, Seoul, Korea; Department of Physical Medicine and Rehabilitation, School of Medicine, University of North Carolina, Chapel Hill, NC, USA; Department of Rehabilitation Medicine, Hanyang University, Seoul and Department of Physical Medicine and Rehabilitation, School of Medicine, University of North Carolina, Chapel Hill, NC, USA

## Abstract

**Objective**: To evaluate the effect of an arm sling on gait speed and energy efficiency of patients with hemiplegia.

**Design**: A randomized crossover design.

**Setting**: A rehabilitation department of a university hospital.

**Subjects**: Thirty-seven outpatients with hemiplegia were included in this study.

**Interventions**: All patients walked on a 20-m walkway twice on the same day, randomly with and without an arm sling, at a self selected speed.

**Main measures**: The heart rate, gait speed, oxygen cost and oxygen rate were measured on all patients. We analysed all values with and without an arm sling and also compared them after all patients being stratified according to demographic and clinical characteristics.

**Results**: When we compared the heart rate between walking with (90.7 ± 17.2 beats/min) and without (91.2 ± 18.6 beats/min) the arm sling, it was significantly decreased while walking with the arm sling. When we compared the gait speed between walking with (32.8 m/min) and without (30.1 m/min), it was significantly increased with the arm sling walking. The O_2_ rate in hemiplegic patients walking with the arm sling was significantly decreased by 7%, compared to walking without arm sling (5.8 mL/kg min and 6.2 mL/kg min, respectively). The O_2_ cost in hemiplegic patients walking without arm sling was significantly 1.4 times greater than walking with it (0.2 mL/kg m and 0.3 mL/kg m, respectively).

**Conclusion**: An arm sling can be used to improve the gait efficiency.

## Introduction

In patients with hemiplegia, gait problems such as poor gait performance, reduced walking endurance and decreased functional mobility are some of the more serious disabilities.^1,2^ Some authors also reported that at a given speed, hemiplegic patients spend more energy per unit of distance travelled than healthy individuals do.^3,4^ In addition, Bohannon *et al.*^5^ reported that most stroke patients rank the restoration of walking in the community as one of the most important goals of rehabilitation.

Hemiplegic shoulder pain is also common after a stroke. It adversely affects the recovery of arm function and independence in the activities of daily living.^6^ Although the relationship between subluxation and hemiplegic shoulder pain remains unclear, various techniques are commonly used to correct this subluxation, including slings^7,8^ and neuromuscular electric stimulation.^9^ Arm slings have various purposes, including realigning scapular symmetry, supporting the forearm in a flexed arm position, improving anatomic alignment with an auxiliary support and supporting the shoulder with a cuff. Despite some uncertainty about their efficacy and timing of use, arm slings are still the most preferred treatment modality for shoulder subluxation in patients with hemiplegia with stroke.^10^

To our knowledge, there has been only one report about the effect of arm sling on gait of hemiplegic patients. Yavuzer and Ergin^11^ reported that an arm sling improved gait pattern using kinematic and kinetic parameters, especially during gait training sessions of patients with hemiplegia who have impaired body image and excessive motion of the centre of gravity. However, no study on the impact of arm sling on energy efficiency in hemiplegia has yet been reported. On the basis of previous study, it is hypothesized that shoulder support by an arm sling improves not only gait pattern but also the energy efficiency of patients with hemiplegia. This study was designed to investigate the effect of shoulder support by an arm sling on gait speed and energy efficiency of patients with hemiplegia.

## Methods

Enrolled subjects were 47 consecutive outpatients with hemiparesis caused by stroke. Inclusion criteria for hemiparetic patients were (a) first cerebrovascular accident verified by computed tomography or magnetic resonance imaging, (b) ability to understand and follow commands, (c) ambulatory before stroke, (d) no medical contraindication to walking, (e) ability to walk independently and (f) below the fourth stage of Brunnstrom stages of motor recovery for the upper extremity. Subjects were excluded if they had visual impairment, premorbid or comorbid neurologic problems other than stroke, or were currently receiving medications known to affect balance or gait, or refused the participation of present study. After exclusion, registered subjects were 37 hemiparetic patients (25 men, 12 women), with an average age of 61.3 years, an average height of 163.1 cm, and an average weight of 60.6 kg.

All hemiparetic patients wore a vest-type shoulder forearm support (Kang’s multi-support sling, Jeonglib O&P, Korea) during the gait trials. The vest-type shoulder forearm support was designed and turned out to prevent glenohumeral subluxation and stabilize the shoulder joint more effectively than a Bobath sling or single strap.^12^

The study was done according to the 1983 revision of the 1975 Declaration of Helsinki. It was approved by the Institutional Review Board of Hanyang University Hospital, and written informed consent was obtained from all patients before data collection.

All subjects were assessed by a single rater for shoulder pain, shoulder subluxation, spasticity and motor recovery in the hemiparetic upper extremity. Shoulder pain was assessed by questioning the subject about whether or not pain was. Diagnosis of glenohumeral subluxation was based on the palpation method.^13^ Evaluation of spasticity in the upper extremity was based on the modified Ashworth Scale.^14^ In addition, upper extremity motor skills were evaluated by using the Brunnstrom stages of motor recovery for the upper extremity.^15^

**Figure 1:**
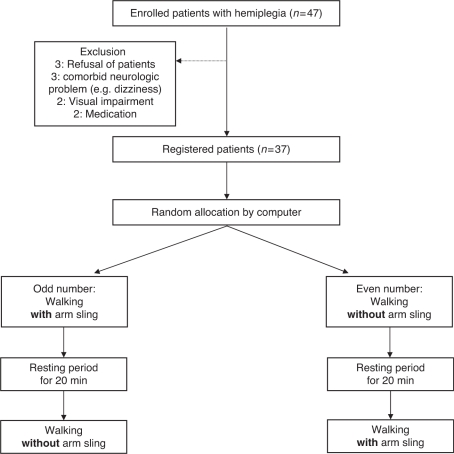
Flowchart of the study.

All the patients walked on a 20-m walkway twice on the same day, randomly with and without an arm sling, at a self-selected speed. Patients were allocated by a computer-generated random sequence provided by a researcher not involved with enrolment. Subjects assigned odd numbers walked with the arm sling first and those with even numbers walked without the arm sling first. After the first walk, patients rested for over 20 minutes; when the rater confirmed that the postwalking heart rate difference from prewalking was less than 5 beats/min, patients started the second walk with the same method. A crossover design was used because a blinded protocol was impractical (Figure 1).

To assess the oxygen cost (O_2_ cost), the patients walked 20 m on level ground at a self-selected comfortable speed. The required time was measured with a stopwatch. Thereafter, we calculated gait speed by dividing 20 m by measured time, and assessed the oxygen rate (O_2_ rate) with a Metamax 3B (Cortex, Germany) portable analyser. The heart rate was monitored before and after walking using a Polar A1 monitor (Polar electro, Finland). The reliability and validity of the tools used in the present study had been reported previously.^16–18^

Data analysis was performed by using SPSS for Windows, version 11.0. Comparisons of heart rate, gait speed, O_2_ rate and O_2_ cost with and without the arm sling were performed by using the non-parametric Wilcoxon signed-rank test. After all patients had been stratified according to their demographic and clinical characteristics, we also compared the same parameters using the non-parametric Mann–Whitney *U*-test.

## Results

Demographic and clinical findings for our patients are shown in Table 1. Heart rate, gait speed, O_2_ rate and O_2_ cost of hemiplegic patients walking with and without the arm sling are given in Table 2.

**Table 1:** Demographics and clinical characteristics of subjects (*n = *37)

Characteristics	No.
Gender: male/female	25/12
Age (years)	61.3 ± 9.3
Height (cm)	163.1 ± 7.3
Weight (kg)	60.6 ± 8.3
Type of stroke: haemorrhage/infarction	12/25
Hemiparetic side: right/left	22/15
Brunnstrom stage of paretic arm: stageI/II/III	7/21/9
MAS in spasticity of paretic arm: range 0–1+/2–4	24/13
Duration (months): <6/≥6	85.8 ± 24.6: 7/30
Shoulder pain: with/without	7/30
Shoulder subluxation: with/without	15/22

MAS, modified Ashworth Scale.

**Table 2:** Comparisons of variables with and without an arm sling at a comfortable pace

	Without arm sling	With arm sling
Heart rate (beats/min)	91.2 ± 18.6	90.7 ± 17.2*
Walking speed (m/min)	30.1 ± 15.5	32.8 ± 14.8*
O_2_ rate (mL/kg min)	6.2 ± 1.7	5.8 ± 1.5*
O_2_ cost (mL/kg m)	0.3 ± 0.2	0.2 ± 0.1*

Values are presented as mean ± standard deviation.

* *P *< 0.05.

The mean and standard deviation of resting heart beat of all patients was 71.3 and 12.5 beats/min. When we compared the heart rate between walking with (90.7 ± 17.2 beats/min) and without (91.2 ± 18.6 beats/min) the arm sling, it was significantly reduced while walking with the arm sling. When we compared the gait speed between walking with (32.8 m/min) and without (30.1 m/min) the arm sling, it was significantly increased with the arm sling. The mean and standard deviation of resting O_2_ rate of all patients was 2.9 and 0.8 mL/kg min. The O_2_ rate in hemiplegic patients with the arm sling was significantly reduced by 7%, compared to walking without the arm sling (5.8 mL/kg min and 6.2 mL/kg min, respectively). The O_2_ cost in hemiplegic patients walking without the arm sling was 1.5 times greater than walking with it (0.2 mL/kg m and 0.3 mL/kg m, respectively).

When patients were stratified according to their demographic and clinical characteristics, such as gender, type of stroke, hemiparetic side, spasticity, motor skill, onset duration, shoulder pain and subluxation, there were no significant differences of heart rate, gait speed, O_2_ rate and O_2_ cost.

## Discussion

Human locomotion involves smooth advancement of the body through space with the least mechanical and physiological energy expenditure,^19^ but the gait of hemiplegic patients with stroke is characterized by asymmetry in stride times and stride length, slow velocity, poor joint and posture control, muscle weakness, abnormal muscle tone and abnormal muscle activation patterns, mostly affecting the paretic side.^20^ Persistent gait deviation increases energy expenditure^21^ and can lead to pain and joint damage.^22^ In addition, in hemiplegic patients, it was reported that the energy expenditure was increased during performance of activities of daily living (ADL) due to the impairment of cardiopulmonary functions, therefore, the cardiovascular loading was also increased.^23^

Given the above background, we speculated that minimization of unnecessary energy expenditure and cardiovascular loading during gait would be important for gait rehabilitation in patients with stroke. Although there has been controversy on the efficiency of an arm sling for treatment of shoulder subluxation or pain, it was reported that an arm sling improved gait pattern.^11^ Therefore, we conducted this study and, to our knowledge, it is the first to report the beneficial effect of an arm sling on the direct energy expenditure in hemiplegic patient while walking.

The measurement of heart rate has been reported to be useful in large studies including the elderly, hemiplegic and cardiovascular patients because it is simple to perform.^24^ In the present study, although mean heart rate was significantly decreased as compared with baseline following the application of an arm sling, it seemed not to be of clinical importance as the difference was too small. However, this tendency implies that the application of an arm sling may reduce the cardiovascular loading during gait in patients with hemiplegia, which also suggests that an arm sling could be an effective tool for gait training in patients with hemiplegia.

Gait speed has a crucial effect on independence in patients with hemiplegia and may vary depending on the authors, but has mostly been reported to be 25–40 m/min.^25,26^ Robinett and Vondron^27^ reported that gait speed enabling independent ADL in healthy people averaged 44.5 m/min. With regard to arm slings, Yavuzer and Ergin^11^ reported that the gait speed and stance period of the paretic side increased, double support time of the paretic side decreased, excursion of the centre of gravity decreased and weight bearing of the paretic side increased in hemiplegic patients while using the arm sling.

In the present study, hemiplegic patients without an arm sling walked on an even level at their comfortable gait speed; the gait speed of this level (30.1 m/min, 1.81 km/h) is similar to slow speed walking (2 km/h) in the previous study.^28^ Although the gait speed following the application of an arm sling was also within slow speed walking level (32.7 m/min, 1.96 km/h), it increased significantly compared with the non-application group. In stroke patients, gait rehabilitation, including increase of gait speed, is essential for the performance of independent ADL. Therefore, the result of the present study suggests that an arm sling may be a useful modalities in gait rehabilitation.

Most of the studies on the effect of supplemental aids on oxygen consumption and energy expenditure have been conducted with a main focus on the lower extremities. Study of the effect of supplemental aids for upper extremities on oxygen consumption and energy expenditure is rare. Hanada and Kerrigan^29^ reported that arm immobilization did not increase energy expenditure during level walking at a comfortable gait speed in a healthy person. On the other hand, Kim *et al.*^28^ reported that arm restriction while walking resulted in significant changes in energy consumption. Oxygen rate with arm restriction was significantly increased during fast walking (6 km/h), but the oxygen consumption rate with arm restriction was not changed while walking at a comfortable speed (4 km/h) and was significantly decreased during slow speed walking (2 km/h). Although subjects were healthy people and arm restriction rather than shoulder support using an arm sling was attempted, these findings were in agreement with the results of the present study that O_2_ rate and O_2_ cost decreased following the application of an arm sling. Because oxygen consumption has been an indicator for measuring the work efficiency and has shown consistent values with no respect to the age, sex and exercise proficiency,^30^ the results of the present study suggest that gait efficiency was significantly improved following the application of an arm sling.

In our clinic, arm slings – usually Kang’s Multi-support – are given to patients with hemiplegia during the flaccid period of the paretic upper extremity. Lee *et al.*^31^ reported that Kang’s Multi-support was helpful in reducing weight asymmetry compared with no sling or a cuff-type sling and that it improved the standing balance of hemiplegic patients because it contacts the patient’s body surface more than a cuff-type sling or no sling. Therefore, we have applied Kang’s Multi-support to hemiplegic patients with stroke to support paretic arm. Because hemiplegic patients with an impaired body image are unaware of the location of their body weight line and they do not have any sense of instability, they fail to make any postural adjustments so the arm sling may serve as a feedback tool and remind the patient’s arm to help postural adjustments. It may also help hemiparetic patients with attention deficit or neglect pay more attention and position the paretic arm correctly.^11,32^

Many therapists do not want hemiparetic patients to use walking aids such as canes or arm slings during daily life because they interfere with functional activities and enhance the flexor synergy of the upper extremity.^11,32^ However, like the previous study by Yavuzer and Ergin, the results of the present study suggest that application of an arm sling affects the gait efficiency in hemiplegic patients positively.

The limitations of this study are as follows. First, the sample size was relatively small. Therefore, the results of present study may not be clinically relevant although they seemed to be a statistically significant. Second, because the present study was conducted for short period, generalization of these results was inconclusive unless further studies on long-term effect of arm sling on hemiplegic gait are performed. However, the present study does use clinical results to suggest that hemiplegic arm support with an arm sling results in improvement of gait in hemiplegic patients. In addition, the present study also provides objective data on arm slings which have been controversial over their usefulness in hemiplegic patients. To clarify the effectiveness of an arm sling for hemiplegic patients, further study considering these limitations is needed.

## Clinical messages

- An arm sling can be used to improve the gait efficiency in hemiplegic patients with stroke.
- The gait speed was significantly increased and the O_2_ rate and O_2_ cost were significantly decreased with the arm sling gait in hemiplegic patients.

## Disclosures

No commercial party having a direct financial interest in the results of the research supporting this article has or will confer a benefit upon the authors or upon any organization with which the authors are associated.
